# Transfer of plantar pressure from the medial to the central forefoot in patients with hallux valgus

**DOI:** 10.1186/s12891-019-2531-2

**Published:** 2019-04-09

**Authors:** Ulf Krister Hofmann, Marco Götze, Katharina Wiesenreiter, Otto Müller, Markus Wünschel, Falk Mittag

**Affiliations:** 1 Department of Orthopaedic Surgery, University Hospital Tübingen, Hoppe-Seyler-Str. 3, 72076 Tübingen, Germany; 2 Department of Orthopaedic and Trauma Surgery, University Hospital Heidelberg, Schlierbacher Landstraße 200a, 69118 Heidelberg, Germany; 3 Orthopädische Gemeinschaftspraxis am Ludwigsplatz, Waldstraße 67, 76133 Karlsruhe, Germany

**Keywords:** Hallux valgus, Pedobarography, Treadmill, Plantar pressure distribution, Gait analysis

## Abstract

**Background:**

The aim of the study was to evaluate changes in plantar pressure distribution in feet affected by hallux valgus compared with their contralateral non-affected feet and with the feet of healthy control subjects.

**Methods:**

Thirty-six patients with unilateral hallux valgus who were indicated for surgery and 30 healthy subjects were assessed on a pedobarographic instrumented treadmill for step length and width, mean stance phase, and plantar foot pressure distribution. Plantar pressure distribution was divided into eight regions.

**Results:**

Significantly higher plantar pressures were observed in hallux valgus feet under the second and third metatarsal heads (*p* = .033) and the fourth and fifth toes (*p* < .001) than in the healthy control feet. Although decreased pressures were measured under the hallux in affected feet (197 [82–467] kPa) in contrast to the contralateral side (221 [89–514] kPa), this difference failed to reach statistical significance (*p* = .055). The gait parameters step width, step length, and single-limb support did not show any differences between hallux valgus and control feet.

**Conclusion:**

Although the literature on changes in plantar pressures in hallux valgus remains divided, our findings on transferring load from the painful medial to the central and lateral forefoot region are consistent with the development of transfer metatarsalgia in patients with hallux valgus.

**Electronic supplementary material:**

The online version of this article (10.1186/s12891-019-2531-2) contains supplementary material, which is available to authorized users.

## Background

While foot pain has always been a highly common symptom among the elderly [1, 2], especially in women [3], the incidence is increasing today, even among younger people [4]. A major cause for these symptoms is hallux valgus deformity, which often leads to severe functional constraints and to transfer metatarsalgia-related forefoot pain. Since the first metatarsophalangeal joint in the physiological situation acts as a pivot for the transfer of body weight during the late stance phase [5], it makes sense that progressive pathological changes, including subluxation in that joint, could interfere with efficient toe-off [6] and thus lead to altered plantar pressure distribution. The way in which these pressures change remains, to date, a matter of debate: Whereas several studies did not detect any significant changes in plantar pressure distribution (reviewed by [6]), two high-quality studies reported increased pressures under the hallux [7, 8] and under the first and second metatarsal heads [7, 9], and one study reported reduced first metatarsal loading [10]. Increased pressures under the small digits (3–5) have also been described [7].

One possible reason for this heterogeneous picture could be the different measurement systems applied: Many pedobarographic or gait analysis studies use single pedobarographic plates [11, 12] or in-shoe pressure measuring systems [13]. Although the plate system offers high spatial resolution, its main disadvantage is the relatively small measuring area, which is especially difficult for fast movement. Longer measurement areas that allow physiological gait can be created with compound measuring plates or carpet walkways; they are, however, costly and remain limited regarding the registered walking distance. In-shoe pressure measuring systems can be worn for a longer time and allow analysis of worn insoles, but their main disadvantage is the relatively low spatial resolution because of the small number of sensors [14]. We therefore used an instrumented treadmill to measure several gait cycles with the patients walking at constant speed. This allows a fluent gait without having to aim for a measuring plate. At the same time, this system offers a reasonable spatial resolution of plantar pressures. Such a set-up has already been used to examine healthy subjects [15] and patients with hallux valgus postoperatively for overall ground-reactive forces [16]. Yet, to our knowledge, no study has examined detailed plantar pressure distribution in patients with hallux valgus by using such an instrumented treadmill.

To better understand changes in plantar pressure distribution could be a valuable key to further improve conservative or surgical therapeutic strategies for patients with hallux valgus. Since the data in the literature with respect to these changes in pressure distribution are still quite heterogeneous, the aim of the present study was to evaluate the effect of hallux valgus deformity on plantar pressure distribution compared with that in healthy feet. We hypothesised that it is possible to differentiate between affected and healthy feet on the basis of these pedobarographic data. In particular, we expected reduced pressures under the medial ray and increased pressures under the second and third metatarsal heads.

## Patients and methods

### Participants

Patients admitted to our department for operative correction of a hallux valgus between August 2010 and February 2012 were asked to participate in this cross-sectional study. Inclusion criteria were radiographically and clinically confirmed and symptomatic hallux valgus with indication for surgery due to experienced pain at the first metatarsophalangeal joint or the adjacent bunion, bursitis or transfer metatarsalgia. Lateral deviation of the hallux only without clinical symptoms was not considered an indication for surgery. Exclusion criteria were prior surgery on the forefoot, pregnancy, no palpable foot pulses, local or systemic inflammation, concomitant cardiopulmonary diseases preventing surgery, a peripheral motor deficit of ≤4 on the scale for muscle power of the British Medical Research Council, a body mass index greater than 35, or an impaired gait for reasons other than the present foot deformity such as osteoarthritis of the knee or a neurological deficit. A control group was formed from healthy volunteers with an American Orthopaedic Foot & Ankle Society (AOFAS) foot score of greater than 90 points without any clinical signs or symptoms of hallux valgus or other pathological conditions of the lower extremities.

Full departmental, institutional, and local ethical committee approvals (project number 122/2012B02) were obtained for this study. Written informed consent was received from all subjects before participation.

### Study design

Pedobarographic assessment was performed by analysing plantar foot pressure distribution, step length and step width, and mean single-limb support. Pain levels were reported on the Visual Analogue Scale and impairment from the hallux valgus was evaluated by using the self-reporting AOFAS foot score. Plain dorso-plantar radiographs of the weight-bearing foot were analysed for hallux valgus and intermetatarsal angles. Passive range of motion in the metatarsophalangeal joint was measured using a standard goniometer.

**Fig. 1:**
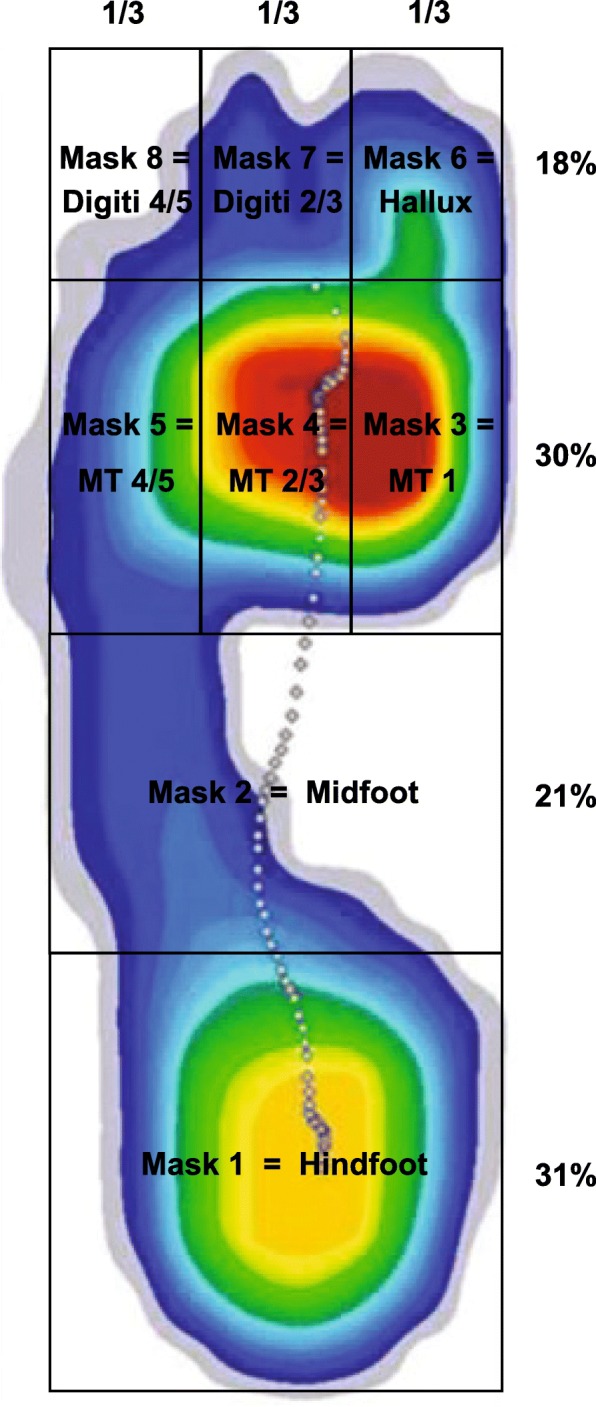
Distribution of the plantar pressure profile into different masks. Longitudinally the foot was subdivided into the hindfoot, midfoot, line of the metatarsal heads, and toe zone. The latter two sections were further subdivided transversally into three equal masks, corresponding to the stronger medial first ray and then the second + third rays and the fourth + fifth rays. Measured pressures are displayed in the form of a heat map, higher pressures indicated by red and lower by blue. The main plantar pressures were measured in the hindfoot region upon heel strike and in the metatarsal region during terminal stance. Abbreviations: MT – metatarsal head

Gait parameters were obtained with the Ergo-Run Medical 8 treadmill (daum electronic GmbH, Fürth, Germany). This treadmill is equipped with the 150 cm × 50 cm electronic sensor mat Gait Analysis System (Zebris Medical GmbH, Insy, Germany) embedded underneath the belt. It contains 10,240 miniature 0.85 cm × 0.85 cm capacitative pressure sensors, registering the exerted force at a rate of 120 Hz and with a measurement precision of 5%, ranging from 1 to 120 N/cm^2^. The speed of the treadmill can be adjusted from 0.2 to 22 km/h at 0.1 km/h intervals. The integrated WinFDM-T software, version 2.0.39 (Zebris Medical GmbH), was used to assess the data. During registration, the patient’s gait was filmed with a Canon MD216 video camera (Canon Inc., Tokyo, Japan) from behind to allow visual verification of the registered parameters with the current gait profile. Mean step width, step length, and single-limb support were automatically reported by the integrated software. For further quantification of the gait profile, we developed a software tool by using MATLAB (MathWorks, Natick/MA, USA) to subdivide the plantar foot measurements of the WinFDM-T software into eight masks: hindfoot, middle foot, first metatarsal head, second and third metatarsal heads, fourth and fifth metatarsal heads, hallux, second and third toes, and fourth and fifth toes (Fig. 1) [17, 18]. In each of these eight regions, peak pressures were registered from heel strike to toe-off during each gait cycle in N/cm^2^. Since it is essential that subjects acclimate sufficiently to the treadmill to be able to obtain comparable data to overground walking [19, 20], all subjects first familiarised themselves with walking on the treadmill until they could comfortably walk without reaching for the handrail at a predetermined study speed of 3.6 km/h and an inclination of 0%. Two measurement runs with six stance phases on each side were measured in all subjects and the corresponding mean values of the peak pressures were calculated.

### Statistical analysis

Categorical variables are described as absolute frequencies. Distributions of variables for all parameters were assessed as histograms. Depending on normality, data are reported as mean (standard deviation) or median (range). Whereas in the study group, the difference between two feet was calculated between the pathological and the healthy foot, in the control group, this difference was formed between the left and right foot. Differences between the healthy and the pathologic foot were calculated by Wilcoxon test, and between the study and the control groups by t-test for independent samples or Mann-Whitney U test as appropriate. For the t-test equality of variances was evaluated with Levene-test. Equal sex distribution between study and control groups was determined by chi-squared test. All reported *p* values have a two-tailed significance level of alpha = 0.05. No adjustment for multiple testing was performed. Statistical analysis was conducted with IBM SPSS, version 22 (IBM, Armonk, NY, USA).

## Results

A total of 62 patients were originally included in the study. After video analysis of the walking profile of the patients, 23 were excluded because they needed to hold the handrail and thus presented with a significantly different gait and pressure distribution profile (data not shown). Another three patients were excluded because of malalignment of the masks by the computer. Therefore, a total of 36 patients (33 women, 3 men) with hallux valgus were assessed and analysed in this study. Their median age was 54 years (range 23–86 years). The left foot was affected in 19 patients and the right foot in 17. The patients presented a median value of 60 (range 0–100) for weight-bearing pain on the Visual Analogue Scale. The mean AOFAS foot score was 54 (SD 10). Some patients (*n* = 17, 47%) used orthopaedic insoles in their everyday lives. The mean hallux valgus angle in the plain dorso-plantar radiographs of the weight-bearing foot was 31° (SD 11°), and the mean intermetatarsal angle was 13° (SD 3°).

The control group, who had no signs of lower extremity pathology, consisted of 19 women and 11 men. They had a median age of 25 years (range 21–61 years) and were significantly younger (*p* < .001) than the study group. Sex was not equally distributed between these two groups (*p* = .005). All control subjects were able to walk freely and barefoot on the treadmill and had no pain at rest or strain whatsoever. The mean AOFAS foot score in healthy subjects was 99 (SD 2). The range of motion in the first metatarsophalangeal joint was 67° (SD 17°) in the hallux valgus group and 97° (SD 21°) in the control group (*p* < .001) (see also Table 1).

**Table 1:** Characteristics of the study and control groups

Variable	Study group	Control group	*P*-value
Men	3	11	**.005** ^a^
Women	33	19	
Age [years]	54 (23–86)	25 (21–61)	**<.001** ^b^
VAS score	60 (0–100)	0	**<.001** ^b^
AOFAS score	53.79 (9.74)	99.2 (1.9)	**<.001** ^c^
Range of motion [^c^]	66.67 (16.86)	97.00 (21.28)	**<.001** ^c^
Hallux valgus angle [^c^]	30.64 (10.66)		
Intermetatarsal angle [^c^]	13.36 (3.13)		

Data are presented as absolute frequencies, median (range), and mean (standard deviation)

Significant *p*-values are denoted in bold

*Abbreviations*: *VAS* Visual Analogue Scale, *AOFAS* American Orthopaedic Foot & Ankle Society

^a^Chi-squared test

^b^Mann-Whitney U test

^c^t-test for independent samples

### Gait analysis

After comparing the macroscopic gait parameters such as step width, step length, and single-limb support, we observed no significant differences between the pathological and the healthy side within the study group or between the study and the control groups (see Table 2). Interestingly, no preferred side was detectable in step length or single-limb support with respect to the affected hallux valgus side: in 15 of 36 patients, the pathological side presented with a shorter step, and in 21 cases, it presented with a longer step. Single-limb support was longer on the affected side in 16 of 36 patients and shorter on that side in 20 patients (see also Table 2).

**Table 2:** Gait analysis

Variable	Study group	Control group	*P*-value
Step width [cm]	9.69 (4.06–16.55)	9.30 (4.21–14.39)	.658^b^
Step length HF [cm]	51.57 (35.60–58.72)	57.03 (49.44–62.48)	
Step length PF [cm]	52.13 (33.13–59.99)	56.85 (49.52–61.65)	
*P*-value	.271^a^	.600^a^	
Difference in step length, PF-HF (study group) or left-right (control group) [cm]	.68 (3.58)	.13 (2.06)	.443^c^
Single-limb support HF [%]	35.46 (33.18–43.29)	35.47 (33.55–37.93)	
Single-limb support PF [%]	35.50 (30.42–40.16)	34.80 (33.71–37.25)	
*P*-value	.626^a^	.074^a^	
Difference in single-limb support, PF-HF (study group) or left-right (control group) [%]	−.35 (1.99)	−.47 (1.19)	.770^c^

The longer step was observed on the pathological side in 21 cases, and the longer single-limb support in 16 of 36 cases. Data are presented as median (range) and mean (standard deviation)

*Abbreviations*: *HF* healthy foot, *PF* pathological foot

^a^Wilcoxon test

^b^Mann-Whitney U test

^c^t-test for independent samples

### Plantar pressure distribution

After analysing plantar pressure distribution, we noted that equal maximum values could be observed in the hindfoot and the midfoot regions on both the valgus side and the healthy side, as well as in the control group. Interestingly, this also applied to the forefoot region (sum of masks 3–8) when we compared the valgus with the healthy side (Table 3). This is consistent with the observation that no difference in step width, step length, or single-limb support could be measured by our set-up. Yet in both feet of the study group, forefoot maximum pressures were significantly higher than in the control group (*p* = .022 pathological foot, *p* = .038 healthy foot).

**Table 3:** Plantar pressures of the different masks of the pathological foot (PF), the healthy foot (HF), and the control group (CG)

Variable	Study group (*n* = 36)		*P*-value^a^	Control group (*n* = 60)	*P*-value^b^ PF-CG	*P*-value^b^ HF-CG
Side	**PF**	**HF**		**CG**		
Hindfoot	243.9 (57.8–359.4)	227.3 (65.4–384.4)	.192	240.4 (80.5–342.3)	.922	.344
Midfoot	103.3 (21.0–162.2)	93.5 (40.0–202.8)	.120	97.4 (39.3–212.9)	.716	.655
MT 1	244.0 (110.4–520.9)	261.8 (150.7–586.7)	.102	257.2 (137.8–522.9)	.669	.565
MT 2/3	338.8 (156.9–666.7)	296.2 (164.7–678.3)	.120	307.0 (208.8–639.0)	**.033**	.591
MT 4/5	202.2 (76.2–418.8)	177.9 (60.3–325.6)	.203	192.6 (85.7–371.5)	.862	.269
Hallux	197.0 (82.1–466.9)	220.7 (89.2–514.2)	.055	203.5 (92.4–488.9)	.159	.472
Digiti 2/3	158.6 (82.1–414.1)	168.0 (90.1–366.3)	.718	146.0 (67.7–334.6)	.183	.259
Digiti 4/5	84.4 (43.7–153.0)	76.9 (38.0–272.3)	.300	59.7 (15.6–138.4)	**<.001**	**.007**
Forefoot (masks 3–8)	409.8 (266.8–666.7)	411.7 (221.7–678.3)	.912	356.2 (233.5–639.0)	**.022**	**.038**

All pressures are reported as kPa. Data are presented as median (range)

Significant *p*-values are denoted in bold

*Abbreviation*: *MT* metatarsal head

^a^Wilcocon test

^b^Mann-Whitney U test

Even more interesting was the detailed mask analysis of the forefoot region: significantly increased pressures could be measured in the region of the second and third metatarsal heads on the valgus side compared with those of the control group (*p* = .033), whereas this was not the case between the healthy foot and the control groups (*p* = .591). As expected, lower maximum pressures were measured under the first metatarsal head of the valgus foot (244 [110–521] kPa) than in the healthy foot (262 [151–587] kPa); the same held true under the hallux mask (valgus foot 197 [82–467] kPa; healthy foot 221 [89–514] kPa). These differences failed, however, to reach statistical significance. Of note, on both sides under the fourth and fifth toes, maximum values were significantly higher than they were in the control group (valgus foot, *p* < .001; healthy foot, *p* = .007). This could indicate an evasive movement where subjects try to lift pressure on both sides off the first ray during the double-limb support phase (Additional file 1: Figure S1).

When measuring foot pressure symmetry between both feet as a parameter, we observed that the greatest differences between the affected and the healthy foot were in the hallux mask (89 [3–351] kPa), under the first metatarsal head (66 [1–213] kPa), and in the mask comprising the second and third metatarsal heads (70 [2–211] kPa) (Table 4). In those areas, the observed asymmetry was also significantly greater than it was in the control group (*p* = .020, *p* = .020, and *p* < .001).

**Table 4:** Absolute differences in plantar pressures between the two feet between the study group and the control group

Variable	Study group (*n* = 36)	Control group (*n* = 30)	*P*-value^a^
Hindfoot	22.1 (6.0–137.5)	20.1 (2.9–94.5)	.634
Midfoot	13.4 (9.0–137.1)	11.0 (2.4–91.5)	.777
MT 1	66.3 (1.3–212.7)	29.1 (3.1–242.5)	**.020**
MT 2/3	70.4 (1.9–210.9)	26.3 (5.0–141.5)	**.001**
MT 4/5	24.2 (1.3–284.7)	33.0 (2.7–136.0)	.571
Hallux	89.1 (3.4–351.1)	35.1 (5.6–236.1)	**.020**
Digiti 2/3	41.8 (1.0–249.6)	33.8 (9.0–167.4)	.503
Digiti 4/5	22.0 (3.0–184.4)	16.0 (1.4–66.0)	.207
Forefoot (masks 3–8)	52.3 (9.0–188.0)	35.5 (3.2–190.1)	.180

Data are presented as median (range). All pressures are reported as kPa

Significant *p*-values are denoted in bold

*Abbreviation*: *MT* metatarsal head

^a^t-test for independent samples

## Discussion

The aim of the present study was to evaluate the effect of hallux valgus deformity on gait and plantar pressure distribution compared with that in healthy feet. For our analyses, we used a treadmill equipped with a sensor mat underneath the belt that allowed for continuous measurements of the walking subjects at a high spatial and temporal resolution.

As described in previous studies, no difference was observed in the spatio-temporal gait parameters such as step width, step length, and single-limb support [6, 9, 21, 22]. The only difference, as observed in one study, was reduced walking speed and overall shorter step length [21]. This did not, however, distinguish between the pathological and the healthy foot of each affected individual - a trait common to many of the previously published studies. When analysing the pathological and healthy side separately in our study, still no difference could be detected in the bilateral parameters of step length and single-limb support. If a difference in plantar pressure distribution exists between hallux valgus and healthy feet, it thus seems to not affect the overall gait pattern. Such a possible difference would thus be mostly compensated within the foot and ankle region itself, without affecting sagittal motion in, for example, the knee or hip region. Indeed, when considering the hindfoot, the midfoot, and the forefoot regions separately, no difference in plantar pressures could be observed between the pathological side and the contralateral healthy foot.

We had hypothesised, nevertheless, that it is possible to differentiate between affected and healthy feet on the basis of these pedobarographic data. We had especially expected reduced pressures under the first ray and increased pressures under the second and third metatarsal heads. To further investigate this possibility, we divided the plantar pressure profile into eight masks of interest, as suggested previously by Hutton et al. [18], and developed a special programme to analyse peak pressures in each of these regions during the gait cycle. Using this set-up, we could identify characteristic differences between hallux valgus feet and healthy control feet: pathological feet showed significantly increased pressures under the second and third metatarsal heads. This was accompanied by reduced pressures under the first ray on the same side, although the measured values failed to reach statistical significance. However, the fact that significantly higher values were again measured under the fourth and fifth toes underlines the concept that, by avoiding the first painful ray, more pressure is transferred laterally, the increased pressures under the fourth and fifth toes indicating a walking profile that is terminally more supine. Even though there is also literature saying that no increased pressure can be measured under the second and third metatarsal head [8, 23] our results are consistent with the common clinical finding of transfer metatarsalgia and hyperkeratosis under the second and third metatarsal heads in patients with hallux valgus. Higher pressure values under the third to fifth metatarsal heads in patients were also described by Hutton et al. [18], who compared the plantar foot pressures of 65 patients with hallux valgus and of 64 healthy subjects by using a single force transducer plate. Mickle et al. and Bryant et al. [7, 9] also found significantly higher peak pressures under the second metatarsal head in patients with hallux valgus. They also showed, however, higher metatarsal head I and hallux peak pressures in patients than in healthy subjects, which contrasts with our findings. In contrast, Hutton et al. [18], described significantly lower peak pressures under the hallux, and lower pressures under the first ray were described by Kadono et al. [10]. After thorough examination of all the studies mentioned, the reasons for this discrepancy in observations remain speculative. It is conceivable that different plantar loading patterns are present at different stages of hallux valgus, as soft tissues adapt to forefoot deformity with bunion formation medially, hyperkeratosis under the second and third metatarsal heads, and different connective tissue tension in the metatarsal head region, with the first metatarsal bone going progressively into increased valgus. Greater hallux valgus severity is correlated with lower pressures under the hallux itself [24]. Yet one would expect that such differences in hallux valgus severity would extrapolate to larger samples. Another relevant factor seems to be the pain level, as individuals affected by pain in the first metatarsophalangeal joint may adopt strategies to offload the painful area during gait [25].

The described differences taken together, as previously suggested by Nix et al. [6], can be attributed to differences in study design; patient-related factors such as age, sex, weight, degree of deviation and pain; and different measuring set-ups. Of interest, however, are the results recently reported by Galica et al. in the Framingham foot study [26]: analysis of plantar pressures of over 3000 participants showed significantly lower maximum forces on hallucal loading and higher forces at the lesser toes in patients with hallux valgus. Moreover, the medial forefoot showed lower maximum forces and peak pressures. The latter differences also failed to be statistically significant. Notably, however, in that model, the authors differentiated only the medial from the lateral forefoot, thus including pressures from the second metatarsal head in the medial ray analysis.

Since it can thus be considered established, that pressures indeed increase under the second and third metatarsal head in patients with symptomatic hallux valgus, an apparent goal of surgery should be to reestablish load bearing on the first ray. If certain procedures do this better than others and if patients also indeed benefit from such a strategy will still need to be further investigated. First studies indicate, however, that patients do clinically benefit from surgical procedures leading to increased plantarflexion with higher postoperative plantar pressures under the first ray [27, 28].

## Study limitations

Although the treadmill set-up offers many advantages, walking in such an artificial environment leads to a slightly altered gait and plantar pressure profile when compared to normal overground walking. A phenomenon observed in several studies is significantly altered kinematics, with the most prominent example being a reduced hip extension and increased hip flexion when walking on the treadmill. The observed differences are, however, usually within a margin of 2–3°, which can be considered negligible [29–31]. Stance time also appears to be slightly reduced [30]. With respect to ground reaction forces, most treadmill parameters seem to be significantly reduced in comparison with overground walking. The observed differences are, however, less than 5%. It has been pointed out, that the magnitude of these differences is comparable to the variability in normal gait parameters and within the range of repeatability and that it should thus not affect basic outcomes [29, 31]. Using a treadmill also leads to a lower spatial resolution in comparison to platform based systems. We also focus on peak pressures in our analysis, force-time integrals were not calculated. Even though measurements were performed video-controlled, the experimental setup did not optoelectronically register additional motion parameters which could have further described the subjects’ gait profile. To be able to measure with the same walking speed in all subjects, we chose a walking speed of 1 m/s, which is a the lower margin of customary speeds for treadmill analyses. While this offers a good comparability with overground walking [32], pathologic conditions might not as easily detected as with higher walking speeds.

Several parameters were significantly different from those of the control group, but were not significantly different from the contralateral side. We cannot evaluate how much of this phenomenon is due to the difference in age between the two groups and how much is due to the fact that hallux valgus is rarely a purely unilateral phenomenon. In many patients, the contralateral side also showed a milder form of hallux valgus, although there was no indication for surgery. For this reason, it is essential to compare the results obtained with those from our healthy control group.

## Conclusion

Using an instrumented treadmill, we measured step width, step length, single-limb support, and plantar pressures divided into eight different masks from 36 patients with hallux valgus, and we compared these values to those from a healthy control group. Although no difference was observed in the spatiotemporal gait parameters, hallux valgus feet showed significantly higher peak pressures under the second and third metatarsal heads and under the fourth and fifth toes. We also observed reduced pressures under the first ray, although they were not statistically significant. The literature in this respect remains divided; nonetheless, this present combination of measured changes is consistent with the development of transfer metatarsalgia in patients with hallux valgus.

## Additional file

Additional file 1: **Figure S1.** Plantar pressures of the different masks in the study and control groups. Plantar pressures are displayed in the form of boxplots. (A) Comparison of plantar pressures between the pathological and the healthy foot in the study group, (B) between the left and right foot in the control group, and (C) between the pathological foot and the control group. (A) Although total forefoot pressures are at similar levels between the pathological foot and the healthy foot of the study group, they are unequally distributed among the masks: pressures are reduced under the hallux valgus in comparison to the healthy side and they are increased under the fourth and fifth toes. Similarly, maximum pressures are reduced under the first metatarsal head but increased under the second and third metatarsal heads. These differences failed, however, to reach statistical significance. Although homogenous pressure distribution was measured between the left and right foot in the control group (B), the supposed differences between the pathological foot and the healthy foot in the study group became more pronounced when the control group was taken as the healthy reference (C): pressures under the pathological foot were significantly increased under the second and third metatarsal heads (*p* = .033) and under the fourth and fifth toes (*p* < .001) in comparison to those of the feet of the control group. Interestingly, forefoot pressures were also significantly increased in both the pathological (*p* = .022) and the healthy (*p* = .038) feet of the study group when compared with those of the control group. All values are reported as kPa. Abbreviations: MT – metatarsal head, Dig – digiti. (PNG 280 kb)

## Abbreviations

AOFAS: American Orthopaedic Foot & Ankle Society
CG: Control group
Dig: Digiti
HF: Healthy foot
MT: Metatarsal head
PF: Pathological foot
VAS: Visual Analogue Scale
