# Supplementary figures and images for: Transfer of plantar pressure from the medial to the central forefoot in patients with hallux valgus
Source: BMC Musculoskelet Disord. 2019 Apr 9;20:149. doi: 10.1186/s12891-019-2531-2 (PMC6454622; doi:10.1186/s12891-019-2531-2)

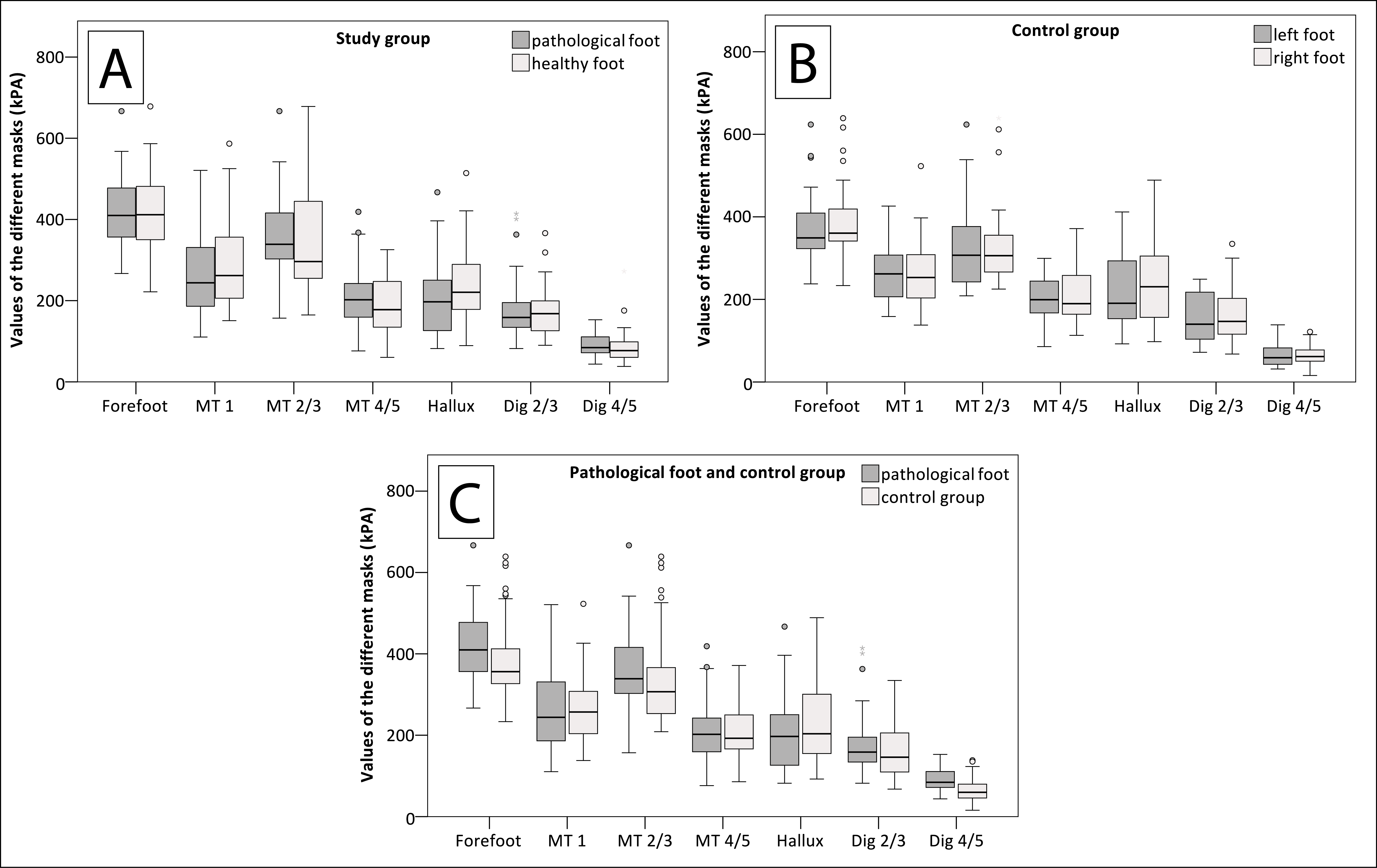

Supplement: Supplementary file 1 — Figure S1. Plantar pressures of the different masks in the study and control groups. Plantar pressures are displayed in the form of boxplots. (A) Comparison of plantar pressures between the pathological and the healthy foot in the study group, (B) between the left and right foot in the control group, and (C) between the pathological foot and the control group. (A) Although total forefoot pressures are at similar levels between the pathological foot and the healthy foot of the study group, they are unequally distributed among the masks: pressures are reduced under the hallux valgus in comparison to the healthy side and they are increased under the fourth and fifth toes. Similarly, maximum pressures are reduced under the first metatarsal head but increased under the second and third metatarsal heads. These differences failed, however, to reach statistical significance. Although homogenous pressure distribution was measured between the left and right foot in the control group (B), the supposed differences between the pathological foot and the healthy foot in the study group became more pronounced when the control group was taken as the healthy reference (C): pressures under the pathological foot were significantly increased under the second and third metatarsal heads (p = .033) and under the fourth and fifth toes (p < .001) in comparison to those of the feet of the control group. Interestingly, forefoot pressures were also significantly increased in both the pathological (p = .022) and the healthy (p = .038) feet of the study group when compared with those of the control group. All values are reported as kPa. Abbreviations: MT – metatarsal head, Dig – digiti. (PNG 280 kb) [file 12891_2019_2531_MOESM1_ESM.png]
